# Benchmark for the Coupled Magneto-Mechanical Boundary Value Problem in Magneto-Active Elastomers

**DOI:** 10.3390/ma14092380

**Published:** 2021-05-03

**Authors:** Philipp Metsch, Raphael Schiedung, Ingo Steinbach, Markus Kästner

**Affiliations:** 1 Institute of Solid Mechanics, Technische Universität Dresden, 01062 Dresden, Germany; philipp.metsch@tu-dresden.de; 2 ICAMS, Ruhr-University Bochum, 44801 Bochum, Germany; ingo.steinbach@rub.de; 3 National Institute for Materials Science (NIMS), Tsukuba 305-0044, Japan; 4 Dresden Center for Computational Materials Science (DCMS), Technische Universität Dresden, 01062 Dresden, Germany

**Keywords:** magneto-active elastomers, strong magneto-mechanical coupling, benchmark

## Abstract

Within this contribution, a novel benchmark problem for the coupled magneto-mechanical boundary value problem in magneto-active elastomers is presented. Being derived from an experimental analysis of magnetically induced interactions in these materials, the problem under investigation allows us to validate different modeling strategies by means of a simple setup with only a few influencing factors. Here, results of a sharp-interface Lagrangian finite element framework and a diffuse-interface Eulerian approach based on the application of a spectral solver on a fixed grid are compared for the simplified two-dimensional as well as the general three-dimensional case. After influences of different boundary conditions and the sample size are analyzed, the results of both strategies are examined: for the material models under consideration, a good agreement of them is found, while all discrepancies can be ascribed to well-known effects described in the literature. Thus, the benchmark problem can be seen as a basis for future comparisons with both other modeling strategies and more elaborate material models.

## 1. Introduction

Multi-physics problems are challenging for numerical solutions due to their inherent non-linearity and the different characteristics of the governing equations for the individual problems [1]. The non-linearity introduced, e.g., by the dependence of material parameters of a special problem on its solution, is usually characterized as *weak* [2], since it will only alter the solution in a monotonous manner. A *strong coupling*, however, arises if multiple fields mutually interact via direct coupling terms within the constitutive equations. Examples of such problems are, among others, a mechanical deformation that is coupled to solute diffusion according to the approach of Larché and Cahn [3], as well as the magnetically induced mechanical interactions in magneto-active elastomers (MAEs) [4,5,6,7]. In both cases, the systems’ behavior not only changes quantitatively, but also qualitatively under the influence of the coupling effects. While, within the first example, the chemo-mechanical coupling yields the phenomenon of inverse ripening that is in contrast to the normal ripening of precipitates in a metallic matrix [8]—see the work of Darvishi Kamachali et al. [9] for more details and a benchmark on this problem—the magneto-mechanical coupling of the second example can cause complex magnetically induced deformations [10,11,12,13] as well as changes of the materials’ stiffness [14,15,16,17]. To this end, the effects emerging from the *strong coupling* of different fields make the treatment of such problems interesting but challenging.

Regarding the solution of strongly coupled problems, numerical simulations usually have to be applied, since analytical expressions can, at best, be found for simplified problems with limited validity or modified versions of the problem using, e.g., the method of manufactured solutions [18]. As the individual problems are highly specialized and, thus, investigated by rather small research communities, they are normally solved using the method that is considered best regarding applicability and performance within these communities. To this end, solution strategies that are well suited for one problem can perform poorly for another one. Revisiting the two examples mentioned before, spectral solvers on regular fixed grids are, e.g., frequently applied to deal with micro-mechanical problems involving a chemo-mechanical coupling, whereas finite element approaches are often used for the solution of magneto-mechanical problems in a finite strain setting.

To facilitate the communication of different research communities, serve as an entry to a specific problem type, and allow for a validation of different modeling strategies, benchmark problems can be of great benefit, as they are often designed to reproduce characteristic mechanisms by means of a rather simple setup [19,20,21]. For multi-physics problems involving strong coupling phenomena, they allow us to analyze and discuss relevant aspects regarding the modeling as well as numerical simulations: How does a specific modeling framework perform compared to other, well-established approaches? Are monolithic or staggered solution schemes preferable to handle the strong coupling? What is the trade-off between accuracy and efficiency? Since the individual problems are too diverse, it is almost impossible to give a conclusive answer to these questions in a general sense. Still, their investigation generates an enhanced understanding of the underlying problem and provides a basis for the exchange of knowledge between different communities.

Within the current contribution, a novel, well-defined benchmark problem for the coupled magneto-mechanical behavior of MAEs is introduced. These materials represent an example of field-controllable functional polymers in which micron-sized magnetizable particles are embedded into a compliant polymer network, see [22,23,24] for a detailed characterization of MAEs. Since the strong coupling of magnetic and mechanical fields allows us to induce mechanical deformations that are significantly larger than magneto-strictive effects observed for single-phase materials [25,26,27], MAEs have attracted considerable research interest in the fields of micro-robots [28] and -pumps [29], as well as coating materials with variable shapes [30]. The ability to control their mechanical modulus using an external magnetic field also allows for technical applications in the areas of actuators and sensors [31,32,33], vibration absorbers [34], as well as prosthetic devices with tunable stiffness [35].

As the modeling of MAEs represents a complex task which requires us to consider physical phenomena across different scales, the proposed benchmark has to allow for a validation of different modeling and simulation strategies by means of a not too complex example, but also needs to be easily extendible to be applicable in situations where, e.g., complex material models are of interest. To this end, the problem introduced here is derived from the experimental analysis of magnetically induced deformations presented in [7]. Using the two-particle system described in the aforementioned work, a detailed analysis of coupling phenomena in MAEs is possible with a manageable number of influencing factors. Here, two extremes regarding the numerical simulation of the problem under investigation are compared with respect to their applicability to reproduce the observed behavior in a simplified setting: an implicit finite element framework based on a monolithic solution of the governing equations as well as a staggered, explicit scheme using a spectral solver on a regular, fixed grid. The organization of the paper is as follows: in Section 2, the benchmark problem is presented—relevant equations are briefly summarized, and the setup of the benchmark is illustrated. The subsequent Section 3 provides an overview of the pursued modeling strategies, while their results for different scenarios are compared in Section 4. Finally, the paper is concluded by a short summary and an outlook to future enhancements in Section 5.

## 2. Benchmark Problem

Within the proposed benchmark problem, the behavior of two magnetizable particles embedded into an elastomer surrounding is analyzed for applied magnetic fields with varying angles. It is based on the study of field-induced interactions in magneto-active elastomers presented in [7] and, thus, originally describes a relevant physical problem that is applicable to validate modeling strategies for MAEs with experimental data. With a primary focus on a systematic comparison of different numerical solution schemes, the problem presented in the following is slightly modified.

### 2.1. Setup

**Figure 1:**
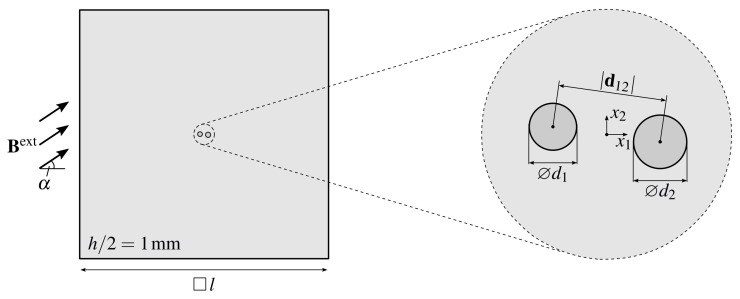
Setup for the proposed benchmark problem: top-view on the center plane of the sample and a magnification of the two magnetizable particles embedded into its center—the global coordinate system is indicated within the magnified area. The problem is symmetric with respect to the depicted $x_{1}$-$x_{2}$-plane and the sample is loaded with an external magnetic field $B^{\mathrm{ext}}$ of varying angle α—for the mechanical boundary conditions, see Section 4.

The geometrical setup of the problem is illustrated in Figure 1: two spherical inclusions of diameters $⌀d_{1}=208\;\mu m$ and $⌀d_{2}=223\;\mu m$ and an initial distance $\left[d_{12}\right]={\left[329\;\mu m,-29\;\mu m,0\;\mu m\right]}^{T}$ are placed into the center of a non-magnetizable elastomer matrix with a quadratic cross section of length *l* and a height of h=2 mm. The choice of the sample size *l* has to ensure that the assumptions of a homogeneous external magnetic field $B^{\mathrm{ext}}$ and vanishing mechanical displacements on the boundary do not affect the particle interactions in the center of the sample. This can be achieved by following the propositions of Bíró and Preis [36] as well as Fetzer et al. [37] and using $l\geq l_{c}$, with $l_{c}$ being the characteristic length of the analyzed structure. Here, the two particles in the center yield $l_{c}\approx 800\;\mu m$, i.e., l≥8 mm. However, the strength of the interaction highly depends on the materials under investigation so that smaller lengths *l* can also ensure unaltered results, see the study on the influence of the sample size performed in Section 4.1. Since both, the geometry of the sample as well as the applied magnetic field, feature a symmetry with respect to the $x_{1}$-$x_{2}$-plane, the computational effort is already reduced in the general three-dimensional case. If the additional assumption of cylindrical inclusions with their cylinder axis along the $x_{3}$-direction is made, a further reduction to a simplified two-dimensional problem is possible.

The external magnetic field $B^{\mathrm{ext}}$ has a magnitude of $B^{\mathrm{ext}}$ = 170 mT, see [7]. After it is applied for an initial angle $\alpha =0{}^{\circ }$, it is rotated clockwise in the $x_{1}$-$x_{2}$-plane while its norm is fixed. For the investigations performed in this contribution, the overall rotation is $180{}^{\circ }$.

### 2.2. Governing Equations

The system under investigation represents a strongly coupled problem, in which effects of the mechanical fields have to be considered in the governing magnetic equations and vice versa. For a detailed presentation of all relevant relations regarding this coupled magneto-mechanical boundary value problem, the authors refer to [13,38,39,40] and references therein—here, only the results of the aforementioned contributions with respect to the equations to be solved are briefly outlined.

By introducing $B_{0}=JF^{-1}\;\cdot \;B$ as well as $H_{0}=F^{T}\;\cdot \;H$ and $M_{0}=F^{T}\;\cdot \;M$ as the reference counterparts of the magnetic induction B, the magnetic field H and the magnetization M, the stationary magnetic part of the coupled problem is given by the Maxwell equations

(1) $$\nabla_{0}\;\cdot \;B_{0}\;=\;0$$

(2) $$\nabla_{0}\times H_{0}\;=\;0$$

and their corresponding jump conditions on a surface of discontinuity [38]. In the pull-back operations above, F and *J* denote the deformation gradient and its determinant, while $\nabla_{0}\left(\;\cdot \;\right)$ in Equations (1) and (2) indicates the Nabla operator with respect to the reference configuration. Since current densities and surface currents are of no relevance for the proposed benchmark problem, they are omitted here. Furthermore, the connection between the magnetic quantities is established via the linking equation

(3) $$B_{0}=\mu_{0}JC^{-1}\;\cdot \;\left(H_{0}+M_{0}\right)$$

with $C=F^{T}\;\cdot \;F$ being the right Cauchy–Green deformation tensor.

Regarding the stationary mechanical part of the problem, the presence of magnetic fields yields additional body force $f^{\mathrm{pon}}={\left(\nabla B\right)}^{T}\;\cdot \;M$ and couple $c^{\mathrm{pon}}=M\times B$ densities [41] which have to be considered within the balances of linear and angular momentum:

(4) $$\nabla \;\cdot \;\sigma +f^{\mathrm{pon}}\;=\;0$$

(5) $$e:\sigma +c^{\mathrm{pon}}\;=0$$

Again, potential mechanical body force densities are of no relevance for the proposed benchmark problem and are thus omitted in Equation (4). Moreover, the third-order tensor e in Equation (5) represents the Levi-Civita, i.e., permutation, symbol, and for the required jump conditions, the authors refer to [39].

### 2.3. Constitutive Behavior

The aim of the original study [7] was a systematic analysis of experimental and theoretical predictions of field-induced interactions in MAEs and, hence, restricted to material models which reproduce the behavior of the individual constituents as closely as possible. However, focusing on a comparison of different numerical solution strategies regarding their performance—especially their results—for a benchmark problem, now allows for a broad range of constitutive models. If the main aspect of the comparison is the question whether different approaches can yield similar or even the same results for a complex numerical problem as, e.g., the coupled magneto-mechanical boundary value problem stated here, the material models can be rather simple. This allows us to compare the numerical frameworks without any additional difficulties that arise if material non-linearities are considered. The benchmark can then be extended to more elaborate constitutive models in order to identify, e.g., implementation errors.

In terms of a first comparison of a Lagrangian finite element framework and a grid-based Eulerian approach using a spectral solver for the solution of the benchmark problem under investigation, an application of linear material models is proposed here. Using the magnetic field H as well as the infinitesimal strain ε as independent variables, this allows for the following formulation of the Helmholtz free energy Ψ:

(6) $$\Psi \left(H,\varepsilon \right)=\Psi^{\mathrm{mag}}\left(H\right)+\Psi^{\mathrm{mech}}\left(\varepsilon \right)=\mu \varepsilon :\varepsilon +\frac{\lambda }{2}\mathrm{tr}{\left(\varepsilon \right)}^{2}-\frac{\mu_{0}}{2}\left(\mu_{r}-1\right)H\;\cdot \;H$$

see [42] for a similar approach using the magnetic induction B as the independent magnetic variable. In Equation (6),

(7) $$\mu =\frac{E}{2(1+\nu )}\;\mathrm{and}\;\lambda =\frac{E\nu }{\left(1+\nu )(1-2\nu \right)}$$

represent Lamé parameters, whereas $\mu_{r}$ denotes the relative permeability of the individual constituents. To obtain the solutions presented in Section 4, the elastomer matrix is characterized by $E^{m}=35\;\mathrm{kPa}$, $\nu^{m}=0.49$ as well as $\mu_{r}^{m}=1$. For the stiff magnetizable particles, $E^{p}=35\;\mathrm{MPa}$, $\nu^{p}=0.3$ and $\mu_{r}^{p}=6$ are applied.

## 3. Numerical Approaches

In the following, the frameworks for the numerical solution of the benchmark are briefly presented to point out characteristics concerning the handling of the strong magneto-mechanical coupling inherent to the problem.

### 3.1. Finite Element Approach

For a solution of the proposed benchmark problem with a continuum-based finite element framework, the equations presented in Section 2.2 are slightly modified. To allow for a simultaneous solution of both Maxwell Equations (1) and (2), the scalar potential φ with H=−∇φ is introduced [43]. Regarding the mechanical part of the problem, an approach using the total stress $\sigma^{\mathrm{tot}}=\sigma +\sigma^{\mathrm{pon}}$ with $f^{\mathrm{pon}}=\nabla \;\cdot \;\sigma^{\mathrm{pon}}$ automatically satisfies the balance of angular momentum [38,39]. To this end, the weak form of the coupled magneto-mechanical boundary value problem is given by:

(8) $$0=\int_{B}B\;\cdot \;\nabla v\;dV+\int_{B}\sigma^{\mathrm{tot}}:\nabla w\;dV$$

with *v* and w being suitable weighting functions for the magnetic and mechanical parts of the problem and B indicating the simulation domain, see [39] for more details on the implementation. Within the finite element simulations, a combined vector $\left\{u,\;\varphi \right\}$ of nodal displacements u and values of the scalar potential φ is solved using a monolithic solution scheme, i.e., the coupling of the magnetic and mechanical subproblems is accounted for with appropriate tangent operators [16].

For the results shown in Section 4, the simulations have been performed using the open-source software tools FEniCS [44] and—for the mesh generation—Gmsh [45].

### 3.2. Grid-Based Spectral Approach

Within the spectral approach, the calculation of the magnetic field and the deformation is separated into two subproblems, which are then solved sequentially. All quantities are discretized on a fixed regular Cartesian grid.

To calculate the magnetic field, a finite difference approach is used to obtain a system of linear equations. Again, a scalar potential φ is used. Applying $H=H^{\mathrm{ext}}-\nabla \varphi$ with the external magnetic field $H^{\mathrm{ext}}=B^{\mathrm{ext}}/\mu_{0}$ and $\nabla \;\cdot \;H^{\mathrm{ext}}=0$, yields

(9) Δφ−∇·M=0

As a linear magnetization behavior is considered here, the magnetic susceptibility $\chi_{v}$ is applied to determine the magnetization via $M=\chi_{v}H$. It is calculated as the weighted average of the particle and matrix susceptibilities $\chi_{v}=\phi \chi_{v}^{p}+\left(1-\phi \right)\chi_{v}^{m}$ using the interpolation function

(10) $$\phi =\left\{\begin{array}{cc} 1 & \mathrm{if}\;r<R-\eta /2\\ \frac{1}{2}-\frac{1}{2}\sin\left(\frac{\pi }{\eta }\left(r-R\right)\right) & \mathrm{if}\;R-\eta /2\leq r\leq R+\eta /2\\ 0 & \mathrm{else} \end{array}\right.$$

in which *R* and η denote the particle radius and the interpolation width, respectively. Inserting the magnetization behavior into Equation (9) gives the relation

(11) $$\left(1+\chi_{v}\right)\Delta \varphi +\nabla \chi_{v}\;\cdot \;\nabla \phi +\nabla \chi_{v}\;\cdot \;H^{\mathrm{ext}}=0$$

for the solution of the scalar magnetic potential φ. It is discretized using a finite difference scheme, and the resulting system of linear equations is solved by applying a biconjugate gradient method [46].

With known magnetic field quantities, the ponderomotive magnetic force density $f^{\mathrm{pon}}$ can be used to determine the deformation within the mechanical subproblem. Therefore, the stress tensor σ=C:ε with C being the linear elastic material stiffness tensor has to be calculated. Using the same weighting procedure as for the magnetization in the magnetic subproblem, i.e., $C=\phi {C\;}^{p}+\left(1-\phi \right){C\;}^{m}$, the equation

(12) $$\nabla \;\cdot \;\left(C:\varepsilon \right)-f^{\mathrm{pon}}=0$$

remains to be solved. This is achieved using an iterative spectral solver—for details on a possible implementation as well as the solver, see [47].

For the results shown in Section 4, the simulations have been performed using the open-source software library Open-Phase [48,49].

## 4. Results

Within this section, results for the proposed benchmark are presented and a comparison of the addressed simulation frameworks is carried out for the general three-dimensional as well as the simplified two-dimensional case. In a first study, the influence of the sample size *l* is analyzed for the chosen set of material parameters. Since solution strategies involving spectral solvers, as the grid-based method introduced in Section 3.2, are preferably applied for problems that can be analyzed using periodicity constraints, the influence of the choice of boundary conditions (BCs) is investigated as well.

### 4.1. Influence of the Sample Size

In the general setup of the proposed benchmark, see Section 2, the sample length *l* has not been specified. Only the restriction that its choice should not alter the particle interactions in the center of the sample was discussed. To this end, *l* is analyzed here in terms of its influence on the resulting change of the inter-particle distance $\Delta d_{12}$ using the finite element approach presented in Section 3.1. For these simulations, only the sample length *l* is varied, while all other parameters remain unchanged.

**Figure 2:**
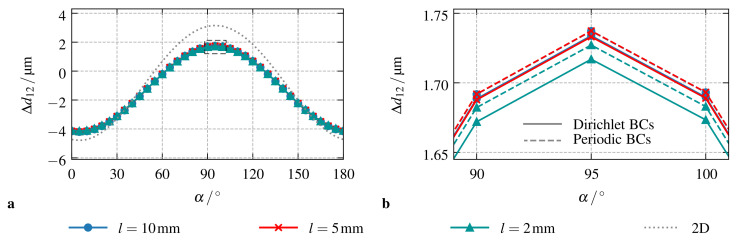
Study on the influence of the sample size. Results obtained using the finite element method for different length parameters *l*: (**a**) Dirichlet BCs according to the setup described in Section 2, and, (**b**) comparison of the results obtained for Dirichlet and periodic BCs close to the peak of $\Delta d_{12}$. The dotted gray lines in (**a**) represent the results for the simplified two-dimensional case.

Figure 2a shows that—if Dirichlet BCs are applied on all outer surfaces of the sample—the length *l* has no influence on the resulting particle interactions. For the investigated set l∈{10,5,2}mm, the predicted change of the inter-particle distance $\Delta d_{12}$ due to the external magnetic field coincides over the whole range of angles α. As already discussed in Section 2, this can be ascribed to the applied material models. Here, linear models for the magnetic as well as the mechanical part of the coupled problem are used: the specific choice of their parameters results in rather weak interactions which rapidly decrease towards to sample boundaries. It is notable that, for the simplified two-dimensional case, the behavior is identical. However, the observed interactions are increased as it is indicated by the dotted gray line in Figure 2a. This is in good agreement with the results of other studies in the field of magneto-active elastomers [7,39].

Regarding the case of periodic BCs, the results are absolutely identical. Here, the external magnetic field is prescribed in a macroscopic, i.e., averaged, sense while periodicity constraints are enforced for the scalar potential as well as the displacements on the sample boundaries. In agreement with the BCs of the original problem, the macroscopic displacement gradient, i.e., the strain, is set to 0. As depicted in the comparison of both loading scenarios in Figure 2b, their deviation is negligible: close to the peak of $\Delta d_{12}$ for $\alpha \approx 95{}^{\circ }$, the difference between simulations performed with l=10 mm using Dirichlet BCs and with l=2 mm applying periodic BCs is less than 0.5.

As a result of this study, the subsequent comparison of the presented simulation frameworks can—for the chosen material models and parameters—be performed for the smallest sample size using periodic boundary conditions without introducing additional boundary effects or changes in the observable coupling phenomena. The computational effort can, thus, be significantly reduced.

### 4.2. Comparison of the Numerical Simulation Frameworks

For the comparison of the finite element and grid-based spectral approaches, the benchmark problem is solved in its modified version: a sample with a quadratic cross section of length l=2 mm is exposed to the external magnetic field $B^{\mathrm{ext}}$, while periodicity constraints are applied for the displacement u as well as the scalar potential φ, see Section 4.1. As both numerical frameworks have already been described in Section 3, only their discretization of the problem remains to be discussed. In order to ensure a good resolution of the simulation domain—especially its center comprising the magnetizable particles—200 equidistant grid points resulting in a grid size of 10 μm are used in each spatial direction within the spectral approach. For the two- and three-dimensional benchmark simulations, this results in a total degree of freedom (DOF) of $1.2\times 10^{5}$ and $3.2\times 10^{7}$, respectively. Within the finite element simulations, only the center of the sample is highly refined, whereas the mesh size is increased significantly towards its boundaries. Here, an exponential function similar to the probability density function of a normal distribution is applied to ensure a number of 54 quadratic tetrahedral elements along the particles circumferences. This results in a minimum mesh size of approximately 12 μm and total DOFs of $1.85\times 10^{4}$ as well as $3.98\times 10^{5}$ for the 2D and 3D problems.

**Figure 3:**
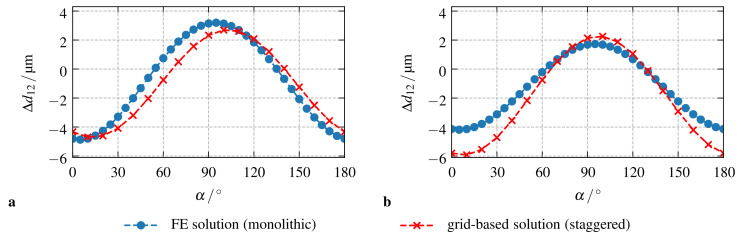
Comparison of the presented numerical frameworks: change of the inter-particle distance $\Delta d_{12}$ in a rotating magnetic field for (**a**) the simplified two-dimensional setup, and, (**b**) the general three-dimensional case. For both cases, the simulations have been performed using periodic BCs and a sample length l=2 mm.

Figure 3a shows the results for the comparison of the two-dimensional setup. It is apparent that both numerical frameworks show a good agreement for all angles α of the external magnetic field—the deviation for their maximum values of $\Delta d_{12}$ is ca. 15. However, a slight shift of the simulation results can be observed: compared to the finite element results, the predictions of the staggered, grid-based approach are delayed by about $\Delta \alpha =5{}^{\circ }$. For the three-dimensional setup depicted in Figure 3b, the situation is different. Here, the staggered, grid-based approach shows increased coupling effects for all angles α of the external magnetic field. While the results of both approaches are in a good qualitative agreement —the slight phase-shift of about $5{}^{\circ }$ is the same as in the two-dimensional problem—their maximum discrepancy is in the range of 40% for $\alpha =10{}^{\circ }$.

**Figure 4:**
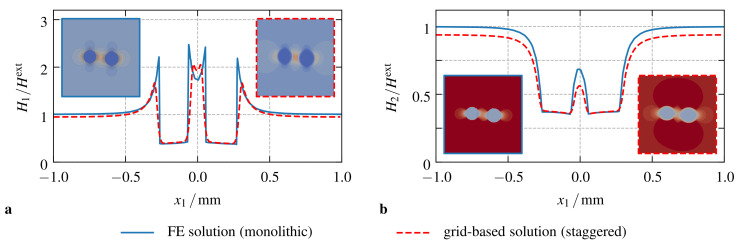
Plot of the normalized magnetic field along the $x_{1}$-axis of the sample: (**a**) results for $\alpha =0{}^{\circ }$, and, (**b**) for $\alpha =90{}^{\circ }$. The embedded surface plots show the distributions of the field within the whole sample using the same scale as indicated in the plots.

In order to assess these differences of the two numerical approaches, a closer look on the course of the magnetic field H along the $x_{1}$-axis of the simulation domain can be helpful. As shown in Figure 4a,b for $\alpha =0{}^{\circ }$ and $\alpha =90{}^{\circ }$, respectively, the normalized magnetic field $H/H^{\mathrm{ext}}$, with $H^{\mathrm{ext}}=B^{\mathrm{ext}}/\mu_{0}$, is comparable for the finite element and grid-based simulation frameworks. Within both materials, the elastomer matrix as well as the magnetizable particles, the course of the field is qualitatively the same—for $\alpha =0{}^{\circ }$ in Figure 4a it almost coincides. As the ponderomotive magnetic body force density $f^{\mathrm{pon}}$ is proportional to the gradient of the magnetic field as well as the magnetization, the magneto-mechanical interaction is restricted to the magnetizable particles. Following the argumentation of Vogel et al. [50], this interaction can be basically accounted to the bulk effect of $f^{\mathrm{pon}}$ within the magnetizable material as well as its jump across material discontinuities. Naturally, the FE approach allows for steep gradients and a sharp transition between the two materials. Compared to that, the diffuse interface of the grid-based approach artificially increases the size of the particles and smoothens the jump across the particle matrix interface. To this end, the bulk effect of $f^{\mathrm{pon}}$ is increased within the grid-based approach, whereas contributions from tractions across the material interfaces are significantly decreased, see Figure 4a,b. This results in the observable differences of the predicted effects for both simulation frameworks and can only be reduced if results with an even higher resolution are compared.

## 5. Conclusions

Within this contribution, a novel, well-defined benchmark for the strong coupling of magneto-mechanical interactions in magneto-active elastomers has been presented. With only minimal adaptations, the proposed problem allows for a comparison of different modeling and solution strategies using a wide range of different material models. Here, the basic setup has been defined and—for the most simple set of material models—a monolithic finite element approach as well as a staggered finite difference framework based on a fixed grid and the application of a spectral solver are investigated with regard to their predictions for the resulting magneto-mechanical interactions. After a thorough analysis of the influence of the sample size and the effect of different kinds of boundary conditions on the results, the performed comparison of these simulation strategies shows a good agreement. Qualitatively, the frameworks yield the same results while all quantitative discrepancies can be ascribed to comprehensible effects that result from the discretization of the problem itself.

With that, the proposed benchmark has proven to be an adequate tool which can serve as an entry to the specific problem of the strong magneto-mechanical coupling in MAEs, but also allows researchers of different communities to exchange their knowledge on various modeling and simulation techniques by means of a rather simple setup with only few influencing factors.

## Data Availability

On inquiry, the data presented in this study is available from the authors.
